# Reduction of Environmental Pollutants and Travel Burden Through an Academic Medical Center-based Electronic Consultation Program

**DOI:** 10.1089/tmj.2023.0435

**Published:** 2024-04-08

**Authors:** Susan L. Moore, Stephanie Grim, Rodger Kessler, Mayra Loera De Luna, Devin E. Miller, John F. Thomas

**Affiliations:** ^1^ Department of Community and Behavioral Health, Colorado School of Public Health, Aurora, Colorado, USA.; ^2^ Peer Mentored Care Collaborative, University of Colorado School of Medicine, Aurora, Colorado, USA.; ^3^ Department of Family Medicine, University of Colorado School of Medicine, Aurora, Colorado, USA.

**Keywords:** *environment*, *emissions*, *telehealth*, *electronic consultation*, *eConsults*, *health care*

## Abstract

**Background:**

*: We evaluated the impact of electronic consultation (eConsult) in reducing the environmental pollutants associated with health care delivery.*

**Methods:**

*: A retrospective analysis of the eConsult data between July 2018 and December 2022 was extracted from the electronic health record (Epic). Travel time and mileage from the patient home to the academic medical center (AMC) were calculated along with fuel expenditure and greenhouses gas savings. Projected savings through the end of the decade were forecast using a random walk model.*

**Results:**

*: A total of 15,499 eConsults were submitted to AMC specialist providers from community primary care providers. Completed eConsults (n = 11,590) eliminated the need for a face-to-face visit with a specialist provider, eliminating mileage, fuel, time, and pollutants associated with face to face visits. In-state travel distance saved was 310,858 miles, travel time saved was 5,491 h, with an associated fuel reduction of 13,575 gallons and $56,893 savings. This reduced greenhouse gas emissions by 128 metric tons of carbon dioxide, 0.022 tons of nitrogen oxide, 0.005 tons of methane, and 0.001 tons of nitrous oxide. Out of state travel distance saved was 188,346 miles with 2,842 h reduced travel time, and associated fuel reduction of 8,225 gallons and of $34,118. Reduced greenhouse gas emissions were equivalent to 77 metric tons of carbon dioxide, 0.0132 tons of nitrogen oxide, 0.0033 tons of methane, and 0.0007 tons of nitrous oxide.*

**Conclusion:**

*: This study indicates that medical care provided through telehealth modalities reduces the environmental impact of pollutants associated with face to face visits.*

## Introduction

Time spent traveling to and from health care clinics, waiting for appointments, consulting with physicians and other providers, and receiving care represents time away from work and other activities, and contributes to a significant burden that often limits patients' access to care. This is further exacerbated for those in rural locations as specialists are often associated with academic medical centers (AMC) located in urban areas. Telehealth is one way to improve access without requiring in-person travel.^1–3^ Electronic consultations (eConsults) represent a method for asynchronous data and communication exchanges between a primary care and specialist provider regarding patient-focused clinical questions. Unlike traditional telemedicine, this model is not an interaction between a doctor and a patient, but rather between two providers to support primary care provider (PCP) management of subspecialty conditions in the primary care setting.

Thus, eConsults serve as a valuable telehealth tool to help build capacity within the primary care medical home by enabling clinical decision support and training for local PCPs who are consequently able to provide a broader range of care for their patients. eConsults also reduce the need for in-person specialty visits, which allows quality health care to be delivered to patients in communities where in-person specialty care is not available and reduces the burden of travel that would be otherwise experienced if patients were required to visit an AMC for in-person specialty care.^4–6^

Although a fair amount of research has documented the environmental impact of reduced travel associated with traditional types of telemedicine,^7–10^ there has not been an evaluation of the benefits of eConsults with regard to travel mileage, travel time, travel cost, and environmental impact. This study examines the impact of an AMC eConsult program in reducing distances traveled by patients and families and the resulting reduction in greenhouse gases associated with this decreased travel.

## Methods

### PROGRAM AND SETTING

Study data were obtained from the eConsult program administered by the Peer Mentored Care Collaborative (PMCC) at the University of Colorado School of Medicine (CUSOM), located on the Anschutz Medical Campus in Aurora, Colorado. The primary focus of the program is to support PCPs across the state to (1) improve access to care for the patients in their practice, (2) integrate best practices in the primary-specialty care interface, (3) decrease overall health care expenditures, and (4) improve overall satisfaction of patients and providers alike. Originating in April 2018, the PMCC eConsult program was modeled after the Association of American Medical Colleges (AAMC)'s Project Coordinating Optimal Referral Experiences (CORE) national program.^11^

The program uses electronic health record (EHR) tools to enhance referrals and manage consult requests and asynchronous communication between PCPs and specialty care providers. The PMCC eConsult program serves a broad base of community-based primary care practices with over 500 individual providers across all Colorado counties, connecting PCPs with specialists in 28 adult clinical practice areas based at the Anschutz AMC. Since inception, the PMCC eConsult program has supported ∼18,000 asynchronous health information exchanges between community-based PCPs and AMC-based specialists.

### STUDY DESIGN AND DATA ANALYSIS

**Fig. 1.**
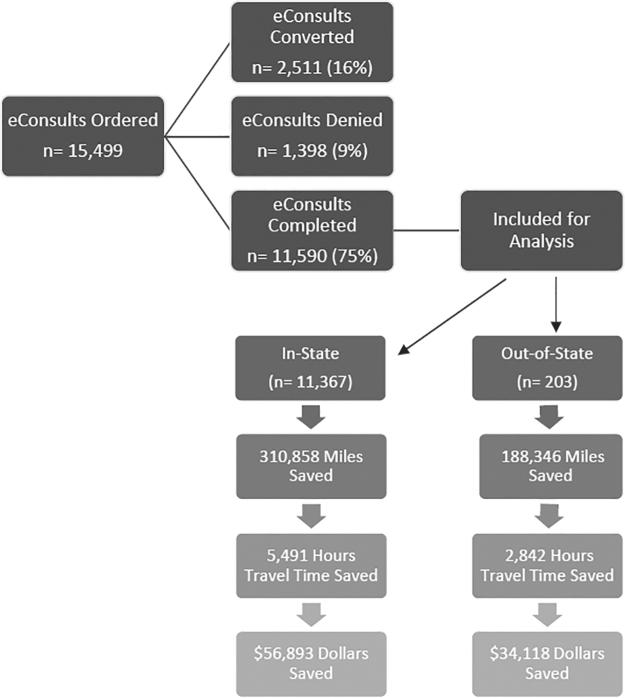
Summary of eConsult analysis.

This retrospective observational study examined patient and administrative data from the CUSOM's EHR (Epic Systems, Verona, WI). All analyses were conducted using RStudio 2022.07.2.^12^ Patient demographic data for eConsult program encounters between July 2018 and December 2022 were extracted from the EHR and analyzed. Each eConsult completed was considered as equivalent to one in-person visit. This equivalence represents adoption of a conservative approach rather than using higher established specialty care referral-to-visit ratios to avoid the potential for overstating outcomes. eConsults which were converted to an in-person specialist visit and eConsults, which were denied as logistical in nature or out of provider scope, were excluded from analysis. *Figure 1* summarizes these results in detail.

A geo-mapping database^13^ containing latitude and longitude locations for the population center of each zip code in Colorado generated an exhaustive list of the distance in miles between each county and zip code in the state of Colorado. Distance saved per visit was calculated as the round-trip distance between the patient's home zip code on file in Epic and the CUSOM AMC zip code.

Travel cost savings were calculated using standard medical mileage rates published by the United States Internal Revenue Service^14^ and applied based on eConsult calendar year. Published rates in cents per mile were multiplied by the distance saved per visit as calculated above then converted to dollars.

Travel time per visit was calculated using Google Maps platform data interfaced with a Visual Basic for Applications program to estimate minutes traveled based on optimized round trip route calculation between each patient's home zip code and the CUSOM AMC zip code.^15^ Minutes of travel were subsequently converted to hours and reported accordingly. Patients with an invalid or missing zip code were excluded from analysis. As eConsults were not limited to patients who were residents of Colorado, travel time was reported separately for out-of-state residents.

Fuel consumption and emission calculations were based on United States (U.S.) Environmental Protection Agency (EPA) assumptions for typical passenger vehicles. Average fuel economy for passenger vehicles, including cars, vans, pickup trucks, and sport utility vehicles, and light trucks, was estimated at 22.9 miles per gallon according to Federal Highway Administration statistics for 2021.^16^ Total greenhouse gas emissions equivalents were calculated for the four most common factors: carbon dioxide (CO_2_), nitrogen oxide (NO_x_), methane (CH_4_), and nitrous oxide (N_2_O) using grams per mile estimates published by the EPA^17,18^ and U.S. Department of Transportation.^19^ Emissions were converted from grams to metric tons for the purpose of reporting.

To estimate potential future emissions savings, we forecast eConsult visit numbers from the beginning of 2023 through the end of the decade. Programmatic annual growth rate to date was calculated as *(x(t)/x0)^(1/t) −1^.* Projected program growth was forecasted using a random walk with drift model. We used the model optimizer within the forecast package of R to choose optimal parameters for our model and the estimated AIC to evaluate model fit. This research was approved by the Colorado Multiple Institutional Review Board as #21-2896.

## Results

**Fig. 2.**
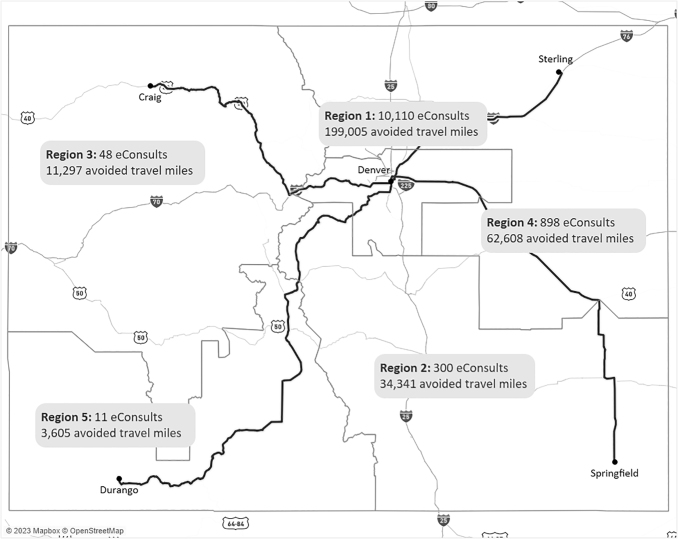
Geographic distribution of eConsults by transportation region. Medium gray lines indicate the borders between the five state transportation regions. Black lines illustrate example highway travel routes between the AMC (Region 1) and four representative cities, one for each of Regions 2–5. AMC, academic medical center.

As shown in *Figure 1*, between July 2018 and December 2022, the program received 15,499 eConsult requests across 28 adult specialty care areas. eConsults were ordered for 12,855 unique patients across 48 of the 64 counties in Colorado. *Figure 2* depicts the distribution of eConsults across the state, aggregated for each of the five transportation regions defined by the Colorado Department of Transportation, along with total mileage saved by region. Example travel routes are also shown to illustrate distances between the CUSOM AMC and representative population centers in each region.

During the study period, 11,590 (75%) of ordered eConsults were completed, 2,511 (16%) were converted to an in-person specialist visit and 1,398 (9%) were denied as they were inappropriate (not enough data, incomplete request, or too complex for an eConsult). Of the completed eConsults, 11,367 were for Colorado residents across 48 counties and 277 zip codes. Two hundred three eConsults were completed for patients from 161 out-of-state zip codes across 34 states. Twenty (0.17%) patients had a missing or invalid zip code on file and were excluded from analysis.

*Table 1* illustrates the reduced travel burden associated with completed in-state eConsults in terms of travel time, total mileage, and dollars saved. The eConsult program resulted in a total travel savings of 310,858 round-trip miles, a total mileage cost savings of $56,893; 5,491 h saved; and fuel savings of 13,575 gallons of gasoline. Average two-way mileage saved was 27.4 miles with a max two-way distance of 501 miles and max travel time of 6.75 h.

**Table 1.** Travel Burden Reduction for In-State eConsults.

IN-STATE TOTALS				
CALENDAR YEAR	MEDICAL MILEAGE REIMBURSEMENT RATE	MILES SAVED THIS YEAR	HOURS SAVED	DOLLARS SAVED
2018	0.18	7,600	131	1,368
2019	0.2	49,738	890	9,948
2020	0.17	76,491	1,404	13,003
2021	0.16	70,790	1,296	11,326
2022	0.2	106,239	1,770	21,248

A breakdown of the reduction in pollutants saved from in-state eConsults demonstrated a total emissions savings of 128 metric tons of CO_2_, 0.001 metric tons of N_2_0, 0.022 metric tons NOx, and 0.005 tons of CH_4_. This is a total savings of 128.03 metric tons of total greenhouse gas emissions.

Completed eConsults for out of state patients saved 188,346 miles and 2,842 h of travel time with an average of 14.0 h of time saved per eConsult. Dollars saved on out of state mileage totaled $34,118. Gallons of fuel saved on out of state travel totaled 8,225. See *Table 2* for full details.

**Table 2.** Travel Burden Reduction for Out-of-State eConsults.

OUT OF STATE TOTALS				
CALENDAR YEAR	MEDICAL MILEAGE REIMBURSEMENT RATE	MILES SAVED THIS YEAR	HOURS SAVED	DOLLARS SAVED
2018	0.18	6,742	100	1,214
2019	0.2	59,905	898	11,981
2020	0.17	64,070	972	10,892
2021	0.16	37,353	561	5,976
2022	0.2	20,276	311	4,055

Out of state eConsults reduced total emissions by 77 metric tons of CO_2_, 0.0007 metric tons of N_2_0, 0.0132 metric tons NOx, and 0.0033 tons of CH_4_. This is a total savings of 77.02 metric tons of total greenhouse gas emissions.

### PROGRAM GROWTH AND PROJECTED IMPACTS

In its initial year, 371 eConsults were ordered through the CUSOM CORE program. By 2022, that number had increased to 4,385, which represents an annual growth rate of 85.4% by year. eConsult volumes forecast from the beginning of 2023 through the end of the decade totaled 42,500. We multiplied the projected number of eConsults by the average two-way travel distance from our existing dataset to estimate the total distance associated with the forecast visits as 1,162,261 miles. We used this number to calculate emissions savings as follows. By the end of 2029, the CORE eConsult program could save several 100 additional tons of emissions, specifically, 478 metric tons of CO_2_, 0.004 tons of N_2_O, 0.081 tons of NOx, and 0.020 tons of CH_4_.

## Discussion

This is the first study to examine the environmental impact of an AMC-based eConsult program. The findings from this work demonstrate multiple positive results, including travel-associated time savings and reductions in number of trips, travel costs, fuel consumption, and emission of pollutants harmful to the environment. The rural and frontier nature of a large swath of Colorado and associated travel distances for in-person specialty visits further contributes to the importance and impact of this type of telehealth model. This urban AMC eConsult program provided care for patients from 75% of Colorado counties, saving some patients 500 miles of travel and up to 7 h of drive time to in-person specialty care appointments on campus.

There are multiple benefits of such a program. Patients can often avoid an unnecessary trip, avoid the need to take time off work or away from other leisure activities, save the time that would otherwise be spent traveling, and reduce their fuel consumption and wear and tear on their vehicle while still receiving the same world class care often only available at an AMC. From a PCP's perspective, providers benefit from the ability to increase their practice capacity, practice at the top of their licensure, and offer better care for more complex patients in their communities.

In addition to the benefits associated with increasing access to care for patients, eConsults hold the potential to contribute to decarbonization and improved environmental stewardship of health care. Health care sector emissions have increased 30% over the last decade and currently account for 9.8% of total national emissions and 4.6% of global emissions.^20^ In light of this impact, the U.S. Department of Health and Human Services has called on health care stakeholders to commit to a 50% reduction in emissions by 2030 and achieve net zero by 2050. State-level policies like those in Colorado accelerate the adoption of clean, renewable resources and energy-efficient planning to preserve the rich natural resources of the state.

These findings demonstrate that yet another sector of the state economy, health care, can contribute in significant ways to cleaner air, reduced greenhouse gasses, and improved public health. Expansion of telehealth thus represents one potential mechanism for direct action by a system to improve health care quality while increasing efficiency and reducing waste to have long-term positive environmental impacts.

This study describes one unique provider group implementing eConsults in the state. As Colorado policy moves to support the expansion of telehealth services such as Medicaid eConsult reimbursement, we anticipate there would be an increase in eConsult adoption across the state beyond the CUSOM CORE eConsult program. An increase in eConsult utilization would lead to greater environmental impacts.

### LIMITATIONS

Our analysis was based on retrospective data, and a prospective analysis would be able to obtain more detailed and nuanced information about additional travel burdens and time impacts associated with face-to-face visits, including factors such as lost wages, waiting time, appointment duration and additional costs such as potential hotel stays, meals away from home, and parking. Next, our calculations assume that all eConsults resulted in comparative benefit attributable to an avoided AMC visit; however, an alternative possibility is that if services were not available from the AMC, patients might have sought care at another location either closer to or further from their home or avoided care entirely. As a result, our estimates may be overvalued or undervalued. We also are unable to ascertain the specific types of vehicle individual patients own, and made assumptions as to the type of transport used.

As Colorado does not have an extensive public transportation system, especially in rural and frontier areas, we assumed that the most likely method of transport was a private passenger vehicle with average emissions. Results may vary by area depending on the prevalence of hybrid or electric cars or the use of older or low mileage cars, or higher use of public transportation. Some of the out of state observations may represent patients whose address of record has not been updated within the EHR or who were visiting Colorado at the time of service. Mileage and travel time calculated for these patients may not truly reflect travel avoided. Future prospective analyses would be well served to take these factors into account. Further studies might also include more detailed economic impact assessments, including information regarding patients' socioeconomic status and household income.

## Conclusions

This study confirms that eConsults reduce the environmental impact of atmospheric pollutants emitted from vehicles by reducing the necessity of individual travel for in-person visits with a specialist across the state of Colorado. Telehealth efforts such as eConsults that support state strategies and polices that seek to create mutual benefits for patients, providers, and the environment hold great promise. eConsults support Colorado's strategic goals of combining health access improvement efforts, affordable care, and environmental justice. This study demonstrates how telehealth efforts support long-term environmentally sustainable options to improve health care access by improving efficiencies and reducing waste. By increasing the use of telehealth across the state, patients and the environment will benefit.
